# The true colours of the Formidable Pygmy Grasshopper (*Notocerus formidabilis* Günther, 1974) from the Sava region (Madagascar)

**DOI:** 10.3897/zookeys.1042.66381

**Published:** 2021-06-07

**Authors:** Éric Mathieu, Marko Pavlović, Josip Skejo

**Affiliations:** 1 Marojejy.com, friends of the Marojejy National Park, Marojejy, Madagascar Marojejy National Park Marojejy Madagascar; 2 Osnovna škola Vidovec, Školska ulica 4, HR-42205 Vidovec, Croatia Osnovna &scaron;kola Vidovec Vidovec Croatia; 3 University of Zagreb, Faculty of Science, Department of Biology, Division of Zoology, Evolution Lab, Rooseveltov trg 6, HR-10000 Zagreb, Croatia University of Zagreb Zagreb Croatia

**Keywords:** Anjanaharibe-Sud, Antohakalava, colouration, *
Holocerus
*, iNaturalist, Madagascar, Marojejy, orange, Tetrigidae

## Abstract

The Formidable Pygmy Grasshopper, *Notocerus formidabilis* Günther, 1974 (Tetrigidae: ‘Malagasy Metrodorinae’), is certainly a stunning, extraordinary insect. Despite the fact that the species was described almost 50 years ago, its beauty had remained completely hidden until recently. The bright yellow colouration of the minute warts on its dorsal hump and even brighter purple-yellowish colouration of its abdomen have been, tragically, completely lost in museum specimens. Luckily, photographs of three live females taken in 2007, 2009 and 2015 were recently uploaded to the iNaturalist platform by the first author of this paper, where they were identified as *N. formidabilis* by the middle and last authors. Along with a male and a female discovered in the MNHN collections (Paris) and the holotype female, these are the only records of the species. All six records are presented and depicted in the present study, and the variation of the species is discussed for the first time. This rare species seems to be endemic to NE Madagascar, a region of truly wonderful diversity.

## Introduction

Most of the biota of Madagascar are endemic to the island, and new and interesting species are being discovered all the time, especially from the northern and north-eastern parts of the island (e.g., Glaw et al. 2021; Moravec et al. 2021). Pygmy grasshoppers are not an exception to this rule. More than 95% of all pygmy grasshopper species known from Madagascar are endemic (Devriese 1991, 1995; Cigliano et al. 2021). The lack of research on Malagasy Tetrigoidea (Günther 1959), combined with the severe deforestation that continuously occurs on the island (Harper et al. 2007; Arias-Ortiz et al. 2021), indicate the necessity of preserving all knowledge on Madagascar’s biodiversity before it is lost. In this short communication, we present the first recent data on the Formidable Pygmy Grasshopper (*Notocerus formidabilis* Günther, 1974), a rare species that has not been observed since its description almost 50 years ago. To date, the only known preserved specimen of the species was the holotype female deposited in Paris. In the present study, we present records of five more specimens, two from the Paris Natural History Museum collection and three based on photographs of live specimens recently uploaded to the iNaturalist platform by the first author of this paper and identified by the middle and last authors. Platforms such as iNaturalist have significantly contributed to the study of biodiversity in recent years (Altrudi 2021; Aristeidou et al. 2021), and faunistic studies have never benefited more from such platforms (e.g., Hochmair et al. 2020; Winterton 2020).

## Materials and methods

All known specimens of *Notocerus formidabilis* were examined by the authors (museum specimens by J. Skejo, live specimens by É. Mathieu in the field and J. Skejo from the photographs). All information relating to these specimens is summarised in Table 1. The holotype of *N. formidabilis* as well as two additional specimens were examined in Paris, while photographs and associated data of three other specimens were uploaded on iNaturalist by É. Mathieu and identified by J. Skejo and M. Pavlović (Table 1). The photographs were taken in 2007, 2009 and 2015 by É. Mathieu during the walks in the mountainous parts of the Sambava district, NE Madagascar (including Antohakalava and Anjanaharibe-Sud). The first photographs were taken in the morning (at 9:12 and 11:04 a.m., respectively), while the third was taken in the afternoon (4:32 p.m.). The specimens were identified by comparison with the holotype and by consulting the original description of Günther (1974). Species mentioned in the discussion were identified using Bolívar (1887), Rehn (1929), Günther (1939, 1959, 1974), and Devriese (1991, 1995). The taxonomy follows the Orthoptera Species File (Cigliano et al. 2021). The common name of the species was introduced by IUCN (Danielczak et al. 2017). Morphological terminology follows Devriese (1991) and Storozhenko and Pushkar (2017). The abbreviation MNHN is used to indicate the Muséum national d’Histoire naturelle in Paris.

**Table 1.** All known records of *Notocerus formidabilis*, listed chronologically from the oldest to the newest. For records from the Muséum national d’Histoire naturelle (MNHN), coordinates were approximated.

Sex and number	Locality and coordinates	Date (time)	Collector or observer	Reference or link
1♀, holotype (MNHN)	Sambava district, Marojejy, Beondroka Mt., 1200 m a.s.l. (14.14S, 49.80E)	VI.1960	Pierre Soga	Günther (1974) (Fig. 1)
1♀, 1♂ (MNHN)	Belanono (= Belalona; 14.48S, 49.92E), between Sambava and Andapa	Probably between 1958 and 1970	André Peyriéras and Jean Vadon	This study (Fig. 2)
1♀	Sambava district, 800–1000 m a.s.l. (14.46S, 49.72E)	19.VIII.2007 (11:04 a.m.)	Éric Mathieu	This study inaturalist.org/observations/70243152 (Fig. 5)
1♀	Sambava district, Antohakalava, 800–1000 m a.s.l. (14.77S, 49.73E)	02.IV.2009 (9:12 a.m.)	Éric Mathieu	This study inaturalist.org/observations/70139087 (Fig. 3)
1♀	Sambava district, Anjanaharibe-Sud special reserve, 800–1000 m a.s.l. (14.73S, 49.56E)	06.V.2015 (4:32 p.m.)	Éric Mathieu	This study inaturalist.org/observations/69859528 (Fig. 4)

### Observation and identification history of *Notocerus formidabilis*

1960 unidentified specimen collected by Soga (deposited in MNHN);

1958–1970 two unidentified specimens collected by Peyriéras and Vadon (deposited in MNHN);

1974 Soga’s specimen described by Günther as a new species, *Notocerus formidabilis*;

2007 unidentified pygmy grasshopper photographed by Mathieu;

2009 second unidentified pygmy grasshopper photographed by Mathieu;

2015 third unidentified pygmy grasshopper photographed by Mathieu;

2016 Peyriéras and Vadon specimens identified as *N. formidabilis* by Skejo;

2021 specimen photographs uploaded to iNaturalist by Mathieu;

2021 Mathieu’s specimens identified as *N. formidabilis* by Skejo and Pavlović.

## Results and discussion

### Family Tetrigidae Rambur, 1838

**Informal group ‘Malagasy Metrodorinae**’

#### Genus Notocerus

Taxon classificationAnimaliaOrthopteraTetrigidae

Hancock, 1900

F2C8A48D-A8BB-5F4F-958F-8FB257CA9E30

##### Type species.

*Notocerus cornutus* Hancock, 1900, by monotypy.

##### Composition and distribution.

This genus includes two species, *N. cornutus* Hancock, 1900 and *N. formidabilis*, both endemic to NE Madagascar.

#### Notocerus formidabilis

Taxon classificationAnimaliaOrthopteraTetrigidae

Günther, 1974

79B06010-0ABB-5E1E-A6AD-44ECD9AD3D4B

##### Type locality.

Sambava district: Marojejy NP: Beondroka Mt., 1200 m a.s.l.

### New records from iNaturalist show species colouration for the first time

**Figure 1.**
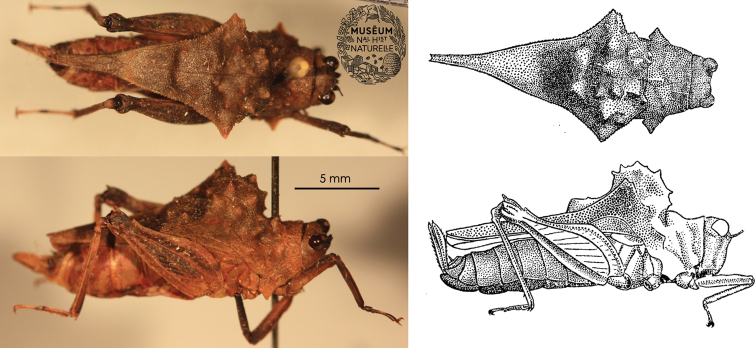
Holotype of the Formidable Pygmy Grasshopper, *Notocerus formidabilis* Günther **A, B** in dorsal view **C, D** in lateral view. Female from NE Madagascar, Sambava-Distrikt, R.N. XII, Marojejy, Beondroka, 1200 m a.s.l., VI.1960, leg. P. Soga **A, C** Josip Skejo and Muséum national d’Histoire naturelle (Paris) **B, D** redrawn after Günther (1974).

**Figure 2.**
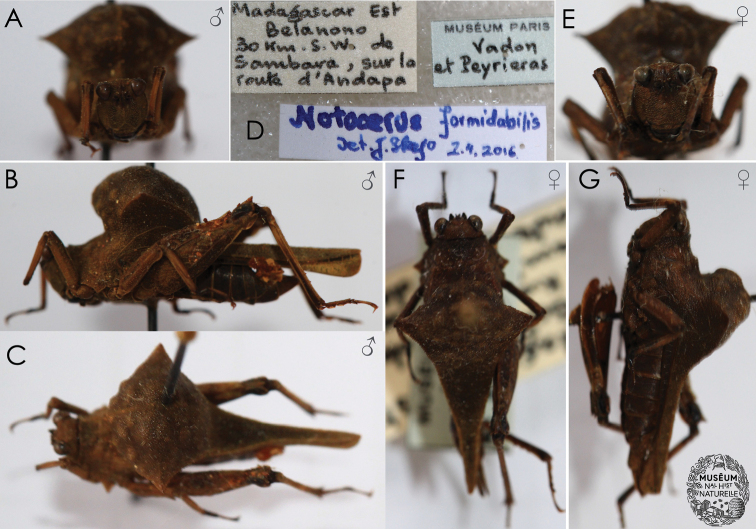
Museum specimens of *Notocerus formidabilis***A–C** male (**A** in frontal view **B** in lateral view, and **C** in dorsal view) **D** labels (same on both specimens) **E–G** female (**E** in frontal view **F** in dorsal view, and **G** in lateral view). Photos by Josip Skejo, Karmela Adžić, Maks Deranja and Muséum national d’Histoire naturelle (Paris).

**Figure 3.**
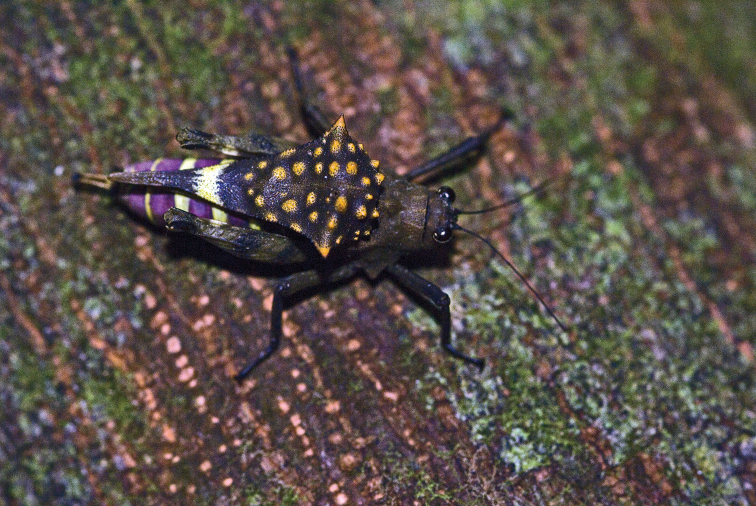
Live female of the Formidable Pygmy Grasshopper, *Notocerus formidabilis*, in dorsal view. Photo by Éric Mathieu.

**Figure 4.**
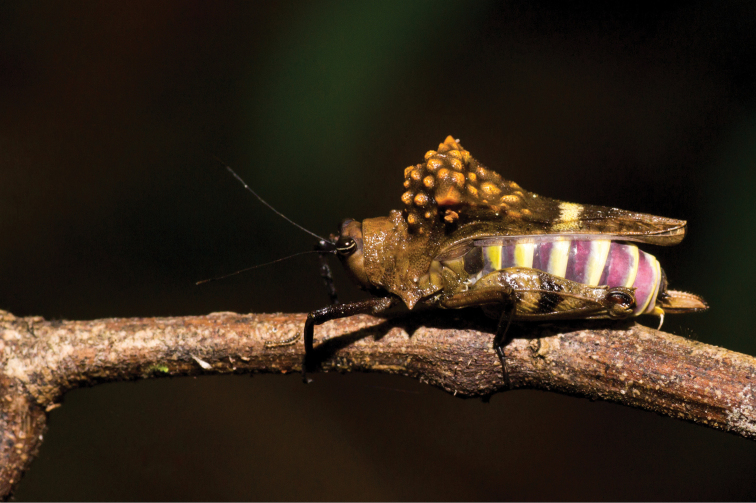
Live female of the Formidable Pygmy Grasshopper, *Notocerus formidabilis*, in lateral view. Photo by Éric Mathieu.

**Figure 5.**
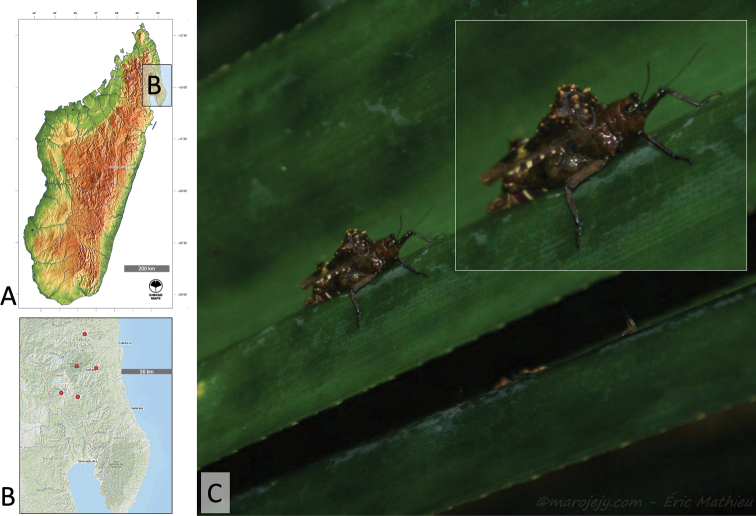
**A, B** Distribution of the Formidable Pygmy Grasshopper and **C** a live female of the Formidable Pygmy Grasshopper, *Notocerus formidabilis*, in lateral view. Map in **A** generated using GinkgoMaps Free Digital Maps, map in **B** using Google maps, and **C** photo by Éric Mathieu.

Without iNaturalist, the collaboration between the authors of this paper would not have been possible, and the photographs may never have been identified to species level. The photos of live specimens of the Formidable Pygmy Grasshopper shown in Figs 3–5 witness how important it is to incorporate *in situ* data into modern biodiversity research. Loss of colouration is known to occur in museum specimens of pygmy grasshoppers (e.g., Mohagan et al. 2020), but it has never been observed to occur to such an extent. Everything we knew about this Malagasy endemic was based on a single known individual of the species, the female holotype (Fig. 1), collected in 1960 in the Sambava district, Marojejy, and deposited in MNHN (Günther 1974). The species was assessed as Near Threatened by the IUCN (Danielczak et al. 2017). Two more individuals (Fig. 2) from Belanono, between Sambava and Andapa, were discovered by the authors in the Orthoptera collections of the MNHN. These were collected between 1958 and 1970 by French naturalists André Peyriéras and Jean Vadon, both of whom worked and lived in Madagascar (Table 1).

### Intraspecific variation

The variation of the specimens reported to date is notable in the shape of the dorsal hump as well as in the shape of the minute warts on the dorsal hump. For example, the holotype female (Fig. 1) has a rough pronotal hump with spine-shaped projections; the two additional specimens from MNHN (Fig. 2) have rather oblique and smooth dorsal humps with small warts; the three specimens reported from photographs (Figs 3–5) have large and rough humps with oblique warts. In the holotype and the other specimens from MNHN, a pale-yellow colouration was observed after careful examination under a stereomicroscope. Therefore, we concluded that the hump seems to be variable in *Notocerus formidabilis* – it can be smaller or larger, more or less smooth, but it is always directed cephalad, and the warts on the hump can be oblique or more or less projected. These differences cannot be attributed to sexual dimorphism, as only one male specimen is reported here (Fig. 2A–C). The variation in pronotal projections among Tetrigidae species has sometimes led to unwarranted new descriptions of the same species, for example in *Trachytettix* Stål, *Cladoramus* Hancock or *Misythus* Stål (Devriese 1999; Cigliano et al. 2021). The Formidable Pygmy Grasshopper is, interestingly, one of a few Tetrigidae species in which the metalateral projections (humeral angles or shoulders) reach more outwards than the apices of the lateral lobes. Examples of other species with very wide humeral projections are *Paragavialidium emeiense* Zheng & Cao (Deng et al. 2012) and *Eufalconius pendleburyi* Günther (Storozhenko and Pushkar 2017) from eastern Asia.

Museum specimens of the Formidable Pygmy Grasshopper are almost uniformly brown (Figs 1, 2), making them so different from live specimens that when the photographs were uploaded by É. Mathieu to iNaturalist in 2021, J. Skejo concluded, at first glance, that they might have represented a new species. The bright orange colouration of the warts on the pronotal hump, together with the bright purple-yellowish abdomen and part of the pronotum, had not been published to date. Only after a careful comparison of the museum specimens and details on their pronotal surface did it become clear that the colour had completely faded. Because of that, for almost 50 years, we were completely deprived of seeing the species in all of its glory. There are still many questions about this species. For example, how did this morphology evolve? Do the yellow warts ‘mimic’ mites? Is the colouration cryptic or aposematic? With this short communication intending to shed some light on the species’ morphology and natural history, we would also like to encourage other researchers to investigate this interesting species if they happen to visit areas in the vicinity of Andapa and Sambava, i.e., the Sava region of NE Madagascar, where the species is endemic (Fig. 5A, B). Similarly to other *Holocerus* species, we expect that this species might be a good flier.

The Sava region, with Marojejy National Park, Anjanaharibe-Sud special reserve and Antohakalava private reserve as the most known reserves, is famous for many animal and plant endemics. For example, the Silky Sifaka (*Propithecus candidus* Grandidier; Mammalia: Primates) (Patel 2014), the Helmet Vanga (*Euryceros prevostii* Lesson; Aves: Passeriformes) (Birdlife International 2018), and the dwarf palm (*Dypsis pumila* Beentje; Plantae: Arecaceae) (Dransfield and Beentje 1995). Now, this region will also be known for one of the morphologically most amazing pygmy grasshoppers.

### iNaturalist contributes to Tetrigidae studies in Madagascar

There are currently 66 iNaturalist records of Tetrigidae from Madagascar, among which 46 (i.e., 70%) are research-grade, altogether representing 16 taxa identified to genus or species level. This number represents around 20% of the known pygmy grasshopper fauna of the island (Cigliano et al. 2021). The Formidable Pygmy Grasshopper is not the only groundhopper species whose first record since its description has been contributed by the iNaturalist platform. Other examples include *Cryptotettix imerina* Rehn, 1929 (‘Malagasy Metrodorinae’) (18.92S, 48.49E, observed by Ehoarn Bidault on 16.IV.2010; inaturalist.org/observations/37580891; and 8.94S, 48.43E, observed by Micha Baum on 25.X.2014; inaturalist.org/observations/53256082), *Eurybiades cerastes* Rehn, 1929 (‘Malagasy Metrodorinae’) (24.46S, 47.01E, observed by Andrianiaina Angelo on 17.XII.2020. inaturalist.org/observations/67190643), *Pterotettix andrei* Bolívar, 1887 (‘Malagasy Metrodorinae’) (14.47S, 49.74E, observed by Éric Mathieu on 30.X.2016.; inaturalist.org/observations/69807072); *Thymochares frontangulus* Günther, 1974 (Cladonotinae) (12.53S, 49.17E, observed by “c_hutton” on 10.III.2019; inaturalist.org/observations/68927516), and *Pseudosystolederus follvikae* Devriese, 1995 (not assigned to any subfamily) (21.26S, 47.43E, observed by Davorka Kitonić and Josip Skejo on 06.I.2019; inaturalist.org/observations/39066871).

## Supplementary Material

XML Treatment for Notocerus

XML Treatment for Notocerus formidabilis

## References

[B1] Altrudi S (2021) Connecting to nature through tech? The case of the iNaturalist app. Convergence 27(1): 124–141. 10.1177/1354856520933064

[B2] Arias-Ortiz A Masqué P Glass L Benson L Kennedy H Duarte CM Garcia-Orellana J Benitez-Nelson CR Humphries MS Ratefinjanahary I Ravelonjatovo J Lovelock CE (2021) Losses of soil organic carbon with deforestation in mangroves of Madagascar. Ecosystems 24(1): 1–19. 10.1007/s10021-020-00500-z

[B3] Aristeidou M Herodotou C Ballard HL Young AN Miller AE Higgins L Johnson RF (2021) Exploring the participation of young citizen scientists in scientific research: The case of iNaturalist. PLoS ONE 16(1): e0245682. 10.1371/journal.pone.0245682 PMC7815142 33465161

[B4] BirdLife International (2018) *Euryceros prevostii*. The IUCN Red List of Threatened Species 2018: e.T22708058A131736320. 10.2305/IUCN.UK.2018-2.RLTS.T22708058A131736320.en

[B5] Bolívar I (1887) Essai sur les Acridiens de la tribu des Tettigidae. Annales de la Société Entomologique de Belgique 31: 175–313.

[B6] Cigliano MM Braun H Eades DC Otte D (2021) Orthoptera Species File. Version 5.0/5.0. http://Orthoptera.SpeciesFile.org [20.03.2021]

[B7] Danielczak A Devriese H Hochkirch A (2017) *Notocerus formidabilis*. The IUCN Red List of Threatened Species 2017: e.T103898756A103900730. 10.2305/IUCN.UK.2017-2.RLTS.T103898756A103900730.en

[B8] Deng WA Zheng ZM Wei SZ Lin MP (2012) A systematic study of the genus *Paragavialidium* Zheng (Orthoptera: Tetrigoidea: Scelimeninae). Zootaxa 3582(1): 48–56. 10.11646/zootaxa.3582.1.5

[B9] Devriese H (1991) Contribution à l’étude des Tetrigidae de Madagascar (Orthoptera). Bulletin et Annales de la Société Royale Belge d’Entomologie 127(5–6): 119–131.

[B10] Devriese H (1995) Deux nouvelles espèces de Tetrigidae de Madagascar (Orthoptera). Bulletin et Annales de la Société Royale Belge d’Entomologie 131(1): 97–105.

[B11] Devriese H (1999) Revision des Xerophyllini d’Afrique (Orthoptera, Tetrigidae). Belgian Journal of Entomology 1(1): 21–99.

[B12] Dransfield J Beentje H (1995) The palms of Madagascar. Royal Botanic Gardens, Richmond, 475 pp.

[B13] Glaw F Köhler J Hawlitschek O Ratsoavina FM Rakotoarison A Scherz MD Vences M (2021) Extreme miniaturization of a new amniote vertebrate and insights into the evolution of genital size in chameleons. Scientific reports 11(1): 1–14. 10.1038/s41598-020-80955-1 33510189 PMC7844282

[B14] Günther K (1939) Revision der Acrydiinae (Orthoptera), III. Sectio Amorphopi (Metrodorae Bol. 1887, aut.). Abhandlungen und Berichte aus den Staatlichen Museen für Tierkunde und Völkerkunde in Dresden (Ser. A: Zool.) (N.F.) 20(1): 16–335.

[B15] Günther K (1959) Die Tetrigidae (Orthopt., Caelifera) von Madagaskar mit einer Erörterung ihrer zoogeographischen Beziehungen und ihrer phylogenetischen Verwandtschaften. Abhandlungen und Berichte aus den Staatlichen Museen für Tierkunde und Völkerkunde in Dresden (Ser. A: Zool.) (N.F.). Ent 24: 3–56.

[B16] Günther K (1974) Beitrag zur Kenntnis der Tetrigiodea (Orth. Caelifera) von Madagaskar und von Mauritius. Bulletin du Muséum national d’Histoire naturelle Paris (Zoologie 3^e^ serie) 236(Zoologie 160): 937–1031.

[B17] Hancock JL (1900) Some new Tettigidae from Madgascar. Occasional Memoirs of the Chicago Entomological Society 1: 1–15.

[B18] Harper GJ Steininger MK Tucker CJ Juhn D Hawkins F (2007) Fifty years of deforestation and forest fragmentation in Madagascar. Environmental conservation 34(4): 325–333. 10.1017/S0376892907004262

[B19] Hochmair HH Scheffrahn RH Basille M Boone M (2020) Evaluating the data quality of iNaturalist termite records. PLoS ONE 15(5): e0226534. 10.1371/journal.pone.0226534 PMC7197788 32365126

[B20] Mohagan AB Leańo EP Melencion MG Patano RP Amoroso V (2020) Yellow Striped Giraffehopper *Spartolus pugionatus* Stål, 1877 comb. resurr. (Tetrigidae: Ophiotettegini) inhabits Mindanao Island of the Philippines’ archipelago. Zootaxa 4722(6): 591–600. 10.11646/zootaxa.4722.6.6 32230602

[B21] Moravec J Brzoska D Vybíral J (2021) New or rare Madagascar tiger beetles – 21. Physodeutera (Microlepidia) propripenis sp. nov., Ph. (M.) marginemaculata (W. Horn) and Ph. (M.) peyrierasi Rivalier (Coleoptera: Cicindelidae). Zootaxa 4941(1): 33–50. 10.11646/zootaxa.4941.1.2 33756947

[B22] Patel ER (2014) Silky Sifaka *Propithecus candidus* Grandidier, 1871. In: Schwitzer C Mittermeier RA Rylands AB Taylor LA Chiozza F Williamson EA Wallies J Clark FE (Eds) Primates in Peril: The World’s 25 Most Endangered Primates 2012–2014.IUCN SSC Primate Specialist Group (PSG), International Primatological Society (IPS), Conservation International (CI), and Bristol Zoological Society, Arlington, 38–43.

[B23] Rehn JAG (1929[1930]) New and little known Madagascar grouselocusts (Orthoptera: Acrididae, Acrydiinae). Proceedings of the Academy of Natural Sciences of Philadelphia 81: 477–519. [5 pls]

[B24] Storozhenko SY Pushkar TI (2017) A new genus of pygmy locusts (Orthoptera: Tetrigidae: Cladonotinae) from the Malay Peninsula. Annales Zoologici 67(1): 47–53. 10.3161/00034541ANZ2017.67.1.006

[B25] Winterton SL (2020) A new bee-mimicking stiletto fly (Therevidae) from China discovered on iNaturalist. Zootaxa 4816(3): 361–369. 10.11646/zootaxa.4816.3.6 33055694

